# 291 Hypernatremia Is Associated with Acute Renal Dysfunction in Severe Burn Patients

**DOI:** 10.1093/jbcr/irad045.266

**Published:** 2023-08-29

**Authors:** Isabel B Obias, Dalton Amador, Amy M Arceneaux, Amina El Ayadi, Georgiy Golovko, Kathleen Karam, Juquan Song, Chris K Soudah, Steven E Wolf

**Affiliations:** The University of Texas Medical Branch, Galveston, Texas; The University of Texas Medical Branch, Galveston, Texas; The University of Texas Medical Branch, Galveston, Texas; The University of Texas Medical Branch, Galveston, Texas; The University of Texas Medical Branch, Galveston, Texas; The University of Texas Medical Branch, Galveston, Texas; The University of Texas Medical Branch, Galveston, Texas; The University of Texas Medical Branch, Galveston, Texas; The University of Texas Medical Branch, Galveston, Texas

## Abstract

**Introduction:**

Hypernatremia often occurs concurrently with acute kidney disease and is associated with increased mortality. It is a common electrolyte disorder presenting in severe burn patients, who are additionally at high risk for acute kidney disease. The purpose of this study is to determine if burn patients presenting with hypernatremia are at greater risk of developing acute kidney disease and related complications.

**Methods:**

Patients diagnosed with severe burns (20% or more of total body surface area) were identified using the TriNetX Research Network and the relevant ICD-10 Codes. TriNetX is a national research database that provides real-time access to de-identified medical records.

Burn patients were stratified into hypernatremic (serum sodium ≥ 145 mmol/L) and eunatremic (serum sodium between 135 and 145 mmol/L) groups. Both groups excluded patients with a prior diagnosis of diabetes or hypothyroidism, as both may render patients more susceptible to electrolyte dysregulation. Patients with a personal history of diseases involving the circulatory, urinary, and respiratory systems were also excluded to control for pre-existing conditions that may affect the outcomes of this study.

We analyzed acute kidney disease, mortality, pulmonary edema, and respiratory failure one month after the onset of hypernatremia; these latter two complications are often associated with kidney injury. Statistical analysis was performed within TriNetX to generate the risk ratios and risk differences for each outcome.

**Results:**

A total of 10,352 patients across 53 healthcare organizations were identified. Excluded from the study were 1,039 (10.04%) patients who were diagnosed with comorbidities. The remaining 9,313 patients were sorted respectively into the hypernatremic (n = 1,116) and eunatremic (n = 6,656) groups. The two cohorts were balanced using (1:1) propensity score matching for age, race, sex, ethnicity, and burn surface area.

The hypernatremic group showed a greater risk of developing acute kidney disease (risk ratio [RR], 4.343, p < 0.001) and associated complications. Severe burn patients with hypernatremia had a 1.987 times greater risk of developing pulmonary edema (p < 0.001), a 2.896 times greater risk of developing respiratory failure (p < 0.001), and a 2.873 times greater 30-day mortality rate (p < 0.001).

**Conclusions:**

Hypernatremia in severe burn patients is associated with an increased risk of developing acute kidney injury, mortality, and related pulmonary complications.

**Applicability of Research to Practice:**

This study indicates that hypernatremia is a potential marker for the development of renal and pulmonary complications.

